# *In Silico* Identification of a Candidate Synthetic Peptide (Tsgf1_18–43_) to Monitor Human Exposure to Tsetse Flies in West Africa

**DOI:** 10.1371/journal.pntd.0002455

**Published:** 2013-09-26

**Authors:** Emilie Dama, Sylvie Cornelie, Mamadou Camara, Martin Bienvenu Somda, Anne Poinsignon, Hamidou Ilboudo, Emmanuel Elanga Ndille, Vincent Jamonneau, Philippe Solano, Franck Remoue, Zakaria Bengaly, Adrien Marie Gaston Belem, Bruno Bucheton

**Affiliations:** 1 Centre International de Recherche-Développement sur l'Elevage en zone Subhumide (CIRDES), Bobo-Dioulasso, Burkina Faso; 2 Institut de Recherche pour le Développement (IRD), UMR MIVEGEC IRD 224-CNRS 5290-Université de Montpellier 1 et Montpellier 2, Montpellier, France; 3 Centre de Recherche Entomologique de Cotonou (CREC), Cotonou, Bénin; 4 Programme National de Lutte contre la Trypanosomose Humaine Africaine en Guinée, Conakry, Guinée; 5 Institut de Recherche pour le Développement, Unité Mixte de Recherche IRD-CIRAD 177, Campus International de Baillarguet, Montpellier, France; 6 Université Polytechnique de Bobo-Dioulasso, Bobo-Dioulasso, Burkina Faso; Lancaster University, United Kingdom

## Abstract

**Background:**

The analysis of humoral responses directed against the saliva of blood-sucking arthropods was shown to provide epidemiological biomarkers of human exposure to vector-borne diseases. However, the use of whole saliva as antigen presents several limitations such as problems of mass production, reproducibility and specificity. The aim of this study was to design a specific biomarker of exposure to tsetse flies based on the *in silico* analysis of three *Glossina* salivary proteins (Ada, Ag5 and Tsgf1) previously shown to be specifically recognized by plasma from exposed individuals.

**Methodology/Principal Findings:**

Synthetic peptides were designed by combining several linear epitope prediction methods and Blast analysis. The most specific peptides were then tested by indirect ELISA on a bank of 160 plasma samples from tsetse infested areas and tsetse free areas. Anti-Tsgf1_18–43_ specific IgG levels were low in all three control populations (from rural Africa, urban Africa and Europe) and were significantly higher (p<0.0001) in the two populations exposed to tsetse flies (Guinean HAT foci, and South West Burkina Faso). A positive correlation was also found between Anti-Tsgf1_18–43_ IgG levels and the risk of being infected by *Trypanosoma brucei gambiense* in the sleeping sickness foci of Guinea.

**Conclusion/Significance:**

The Tsgf1_18–43_ peptide is a suitable and promising candidate to develop a standardize immunoassay allowing large scale monitoring of human exposure to tsetse flies in West Africa. This could provide a new surveillance indicator for tsetse control interventions by HAT control programs.

## Author Summary

Increasing interest is paid to blood-sucking arthropod's salivary antigens to develop host direct biomarkers of exposure. Nevertheless use of whole saliva is problematic both because of mass production and specificity issues. Here, we describe an *in silico* approach we used to identify potential epitopes on the amino acid sequence of three tsetse salivary proteins (Ada, Ag5 and Tsgf1) that were previously shown to be specifically recognized by antibodies from exposed individuals. Three candidate peptides were synthesized and evaluated on a set of plasma collected in different tsetse-infested and tsetse-free areas. The Tsgf1_18–43_ synthetic peptide appeared as a promising candidate to assess human exposure to tsetse flies as antibody responses were low in all three control groups and were significantly higher in our two exposed groups. Significantly higher anti- Tsgf1_18–43_ responses were also observed in sleeping sickness patients as compared to uninfected controls suggesting that Tsgf1_18–43_ may be used both to assess human tsetse contacts and the risk of infection by trypanosomes. This new sero-epidemiological tool could thus help National Control Programs to quickly map human exposure levels in order to better target vector control efforts and monitor vector control efficiency.

## Introduction

With less than 10 000 reported cases in 2009 across Africa [1], the goal of elimination of human African trypanosomiasis (HAT), caused by *Trypanosoma brucei gambiense* (*T. b. gambiense*) and *T. b. rhodesiense* and transmitted by tsetse flies, seems again to be in sight [2]. Active surveillance by medical surveys, during which mass screening of the population is made to identify and treat infected persons in order to reduce the human reservoir, were shown to be effective and resulted in a 69% reduction in the number of new case during the period 1997–2006 in *T. b. gambiense* endemic areas. Out of the 36 endemic countries, 20 are now close to achieving the target of reporting no new cases and eight reported <100 new cases per year [3]. Nevertheless it is also known that this strategy becomes less effective when disease prevalence is becoming low, both because of the weariness from endemic populations leading to dramatic decrease in medical survey attendance [4], and because of cost related issues as the cost to diagnose a patient becomes prohibitive. In the absence of a vaccine or prophylactic molecules for mass treatment, vector control thus appears as an important complementary strategy to reach the goal of elimination or at least a sustainable control of HAT [5]. With the Pan African Tsetse and Trypanosomiasis Eradication Campaign (PATTEC), large scale tsetse eradication campaigns are now underway in several African countries such as in Uganda and Ethiopia in East Africa and Ghana, Burkina Faso and Senegal in West Africa in order to improve the breeding and agricultural potential of these animal African trypanosomiasis (AAT) endemic areas [6]. Vector control campaigns are now progressively extending to HAT endemic areas in combination with medical surveys such as in the Boffa focus along the Guinean coast [7].

Although a diversity of methods are now available to control tsetse populations such as ground and aerial spraying of insecticides, live-bait technologies, *insecticide*-treated targets/traps and use of the sterile insect technique [8], a major challenge still resides in the evaluation of their efficacy and the definition of pertinent indicators to measure intervention successes. Currently entomological evaluation is performed through the monitoring of sentinel traps to measure the apparent tsetse flies density per trap and per day and thus to determine the reduction ratio compared to starting densities [9]. This entomological method nevertheless presents a number of important limitations, namely low sensitivities or efficiencies, as it was shown that only 20% of the traps attracted flies are actually captured [10], [11]. In addition it has been suggested that traps become less efficient when tsetse densities decrease [12] possibly due to density-dependant dispersal [13]. They may thus lead to an underestimation of the true densities. Furthermore, tsetse density itself only poorly reflects the intensity of contact with human populations which is the most crucial parameter when the objective of vector control is to reduce human tsetse contacts to levels incompatible with maintenance of the parasite life cycle [7], [14], [15].

The fact that exposed hosts develop an antibody response against the saliva of blood sucking arthropods has provided a new way to assess individual host exposure to a number of important vectors such as mosquitoes [16]–[18], ticks [19], [20], triatomines [21] or sand flies [22]. Accordingly, we were able to show, in a recent study, that individuals from tsetse infested areas displayed significantly higher antibody titers against whole saliva extracts from *Glossina palpalis gambiensis* (*G. palpalis gambiensis*), the main vector of *T. b. gambiense* in West Africa, as compared to unexposed individuals [23]. Nevertheless, the use of whole saliva extracts is also associated with mass production associated issues that preclude their use for large scale studies [24]. Furthermore, some salivary antigens are conserved and common among the different species of blood sucking arthropods, inducing cross-reactions and loss of specificity when whole saliva extracts are used in immunoassays [25]. To overpass these problems, salivary recombinant proteins have been used to develop standardized epidemiological serological tools to assess exposure to ticks [26], [27], sand flies [28], [29], triatomines [30] or to the afrotropical malaria vectors [31]. Another approach, less demanding in terms of laboratory facilities and well adapted to the environment of research laboratories in Africa, has been to identify specific and immunogenic peptides from the salivary proteins sequences available in the data banks. This strategy was successfully applied to develop serological markers of human exposure to *Aedes aegypti* [32] and *Anopheles gambiae* [33], [34].

In this paper we describe an “in silico” approach, in which several epitope prediction and protein conformation software's were used in combination with blast analyses to define synthetic peptides to assess human exposure to tsetse flies in West Africa. For this study we included three proteins sequences that were shown to be specifically recognized by sera from individuals exposed to tsetse flies [23]: adenosine deaminase (Ada); Antigen 5 (Ag5) and Tsetse Saliva Growth Factor1 (Tsgf1). Most antigenic and specific predicted peptides were then synthesized and tested against a bank of plasma samples collected in populations exposed or not to tsetse flies.

## Materials and Methods

### Ethics statement

Human plasma samples were collected from tsetse infested areas from Guinea and Burkina Faso and tsetse-free areas from Burkina Faso (Bobo-Dioulasso), Southern Benin and France. All samples, used in previously published studies [23], [32], [35], were collected according to the ethical principles of the Helsinki Declaration and were anonymized. All samples collected in Guinea and Burkina Faso were collected during medical surveys led by the National Control Programs of these two countries according to the respective national HAT diagnostic procedures. All participants were informed of the objectives of the study in their own language and signed a written informed consent form. These samples are part of a larger project aiming to improve HAT diagnosis for which approval was obtained from the WHO Research Ethics Review Committee (RPC222) and from the IRD ethical committee (December 2007). Samples from Benin were part of a study on *Aedes aegyti* exposure [32] which was approved by the National Ethical Committee of Benin (IRB 00006860) and the IRD ethical committee (April 2008).

### Human plasma

A total of 160 samples were included for the purpose of this study as follows:

#### Guinean HAT foci

80 plasma samples, collected during medical surveys performed by the National Control Program of Guinea (mean age = 35.4 years old [5–80]) in the Forecariah and Dubreka mangrove HAT foci [23], [35] that are currently the most active foci in West Africa. The Guinean mangrove ecosystem harbors high densities of *G. palpalis gambiensis* [7] and humans living in these areas are in close contact with tsetse flies during their daily activities [36]. Thirty six samples were from individuals diagnosed as HAT patients, and 44 were from uninfected individuals sampled in the same villages.

#### Batié (Burkina Faso)

10 samples collected from villagers (mean age = 32 years old [7–78]) during a medical survey led by the National Control Program of Burkina Faso in 2008. Batié is located in South-West (SW) Burkina Faso in a HAT historical focus where tsetse flies and animal trypanosomiasis are still present [37]. In SW Burkina Faso, three major tsetse species can be encountered. *G. palpalis gambiensis* and *G. tachinoides* are riverine species found in forests galleries along rivers. *G. morsitans submorsitans* is a savannah fly that is progressively disappearing due to the reduction of the wild life fauna and to the degradation of its habitat in relation with increasing human environmental pressure, and is now largely restricted to protected areas [38]. No HAT cases have been diagnosed in this area during a survey during which 4531 individuals were screened [39].

#### Bobo-Dioulasso (Burkina Faso)

17 samples collected from citizen volunteers (mean age = 24.8 years old [18]–[33]) that did not report travelling outside of the city for at least three months [23]. Bobo-Dioulasso is the second city in Burkina Faso and is considered free of tsetse flies. Inhabitants are nevertheless exposed to a number of other biting arthropods such as C*ulex* mosquito species, *Anopheles gambiae* s.l., *Anopheles arabiensis* [40] or sandflies. Tsetse flies are nevertheless present in sites that are in the neighborhood of the city and we cannot exclude that some study subjects may have experienced occasional exposure while visiting these sites. Samples were included as our “urban African unexposed group”.

#### Southern Benin

31 samples collected during a longitudinal survey of a cohort of children aged between 1 and 5 years old conducted between February 2008 and October 2009 in 7 villages of the Kpomasse-Tori Bossito health district in southern Benin as detailed elsewhere [32]. In this area of southern Benin where riparian forests are highly degraded and only few sacred wood persist due to the intense agricultural activities led in the area, the presence of tsetse flies is very unlikely. Nevertheless, although study samples were chosen in villages located away from river sites and all were from young children, as in Bobo-Dioulasso we cannot however exclude that some study subjects may have been occasionally bitten by tsetse flies. Samples were included as our “rural African unexposed group”.

#### Bordeaux (France)

As a “non African unexposed group” we also included 22 samples collected from adult blood donors of the French blood bank (Etablissement Français du Sang) in Bordeaux.

### Peptide identification

Bioinformatics analyses were carried on the mature protein sequences (signal peptide removed) of the *Glossina* Ada (D3TR66), Tsgf1 (Q9U7C6), and Ag5 (D3TMF1) salivary proteins that were previously shown to react specifically with plasma from tsetse infested areas [23]. Protein sequences were from *G. morsitans morsitans* as they are currently the only available tsetse sequences in the data banks. In a first step, each of the protein sequences was sequentially submitted to an array of four different epitope prediction algorithms. The identification of putative linear B-cell epitopes was performed on the NETSurfP [41], ABCpred [42] and Bcepred [43] servers using the B cell epitope database BCIPEP [44], which contains 3 031 entries that include 763 immunodominant, 1 797 immunogenic, and 471 null-immunogenic epitopes [45]. MHC class2 binding regions were also searched with the Proped-2 online service [46]. All epitopes (5–6 amino-acid length) that were identified by at least three out of four algorithms were selected for further analyses. In a second step, 10 amino acid sequences were added on each side of the selected epitopes and corresponding sequences were blasted on all non-redundant Genbank CDS databases [47]. After this step the best candidate peptide (with highest specificity to *Glossina*) was selected for each of the study salivary antigens (Ada, Ag5 and Tsgf1). Accessibility of candidate peptides at the surface of the protein was then checked by performing 3D structure models of the proteins with Phyre [48] and verified with the molProbity server [49]. Peptides were synthesized by the Genepep Company (Saint-Jean de Vedas, France) with a purity control by HPLC>94%. All peptides were shipped lyophilized and were resuspended in ultra-pure water and then frozen in aliquots.

### Peptide immunoassays

ELISA (Enzyme Linked ImmunoSorbent Assay) was used to measure the peptides specific IgG responses in human plasma. Maxisorp plates (Nunc, Roskilde, Denmark) were coated for 2 h 30 min at 37°C with peptides (20 µg/ml) diluted in carbonate/bicarbonate buffer. After washing with PBS tween 0.1%, plates were saturated 1 h with the TBS-protein free Blocking Buffer (Pierce, Rockford, IL, USA). Plasma diluted in PBS-Tween 1% (1∶10 for Ada_188–213_ and Ag5_105–130_, 1∶40 for Tsgf1_18–43_) were incubated overnight at 4°C. Anti-human IgG biotinylated antibodies (BD Pharmingen, San Diego, CA, USA) diluted 1/1000 in PBS Tween 1% or in Blocking Buffer (for Ada_188–213_), were incubated for 1 h at 37°C. This step was followed by 1 h incubation with streptavidine coupled with peroxidase (1∶1000) (GE healthcare biosciences, Uppsala, Sweden). The colorimetric development was done with ABTS (2,29-azino-bis (3-ethylbenzthiazoline 6-sulfonic acid), Sigma, St Louis, MO, USA) diluted in 50 mM citrate buffer (pH 4) containing 0,003% of H_2_O_2_. The optical density was measured at 405 nm. Each plasma sample was tested in duplicate and once in a well without antigen. The individual results were expressed in ΔOD measured according to the following formula: ΔOD = ODx–ODn where ODx is the average of the duplicate wells and ODn the optical density of the well without Ag. When ΔOD values were negative ΔOD were considered to be 0.

### Statistical analysis

Statistical analyses were carried out with the GraphPad Prism software (San Diego, CA, USA). After verifying that the values did not assume a Gaussian distribution, the Mann Whitney U nonparametric test was used to compare IgG levels between two independent groups and the Kruskal-Wallis nonparametric test for comparison between more than two groups. Differences were considered significant for p<0.05.

## Results

### Peptide design

**Figure 1:**
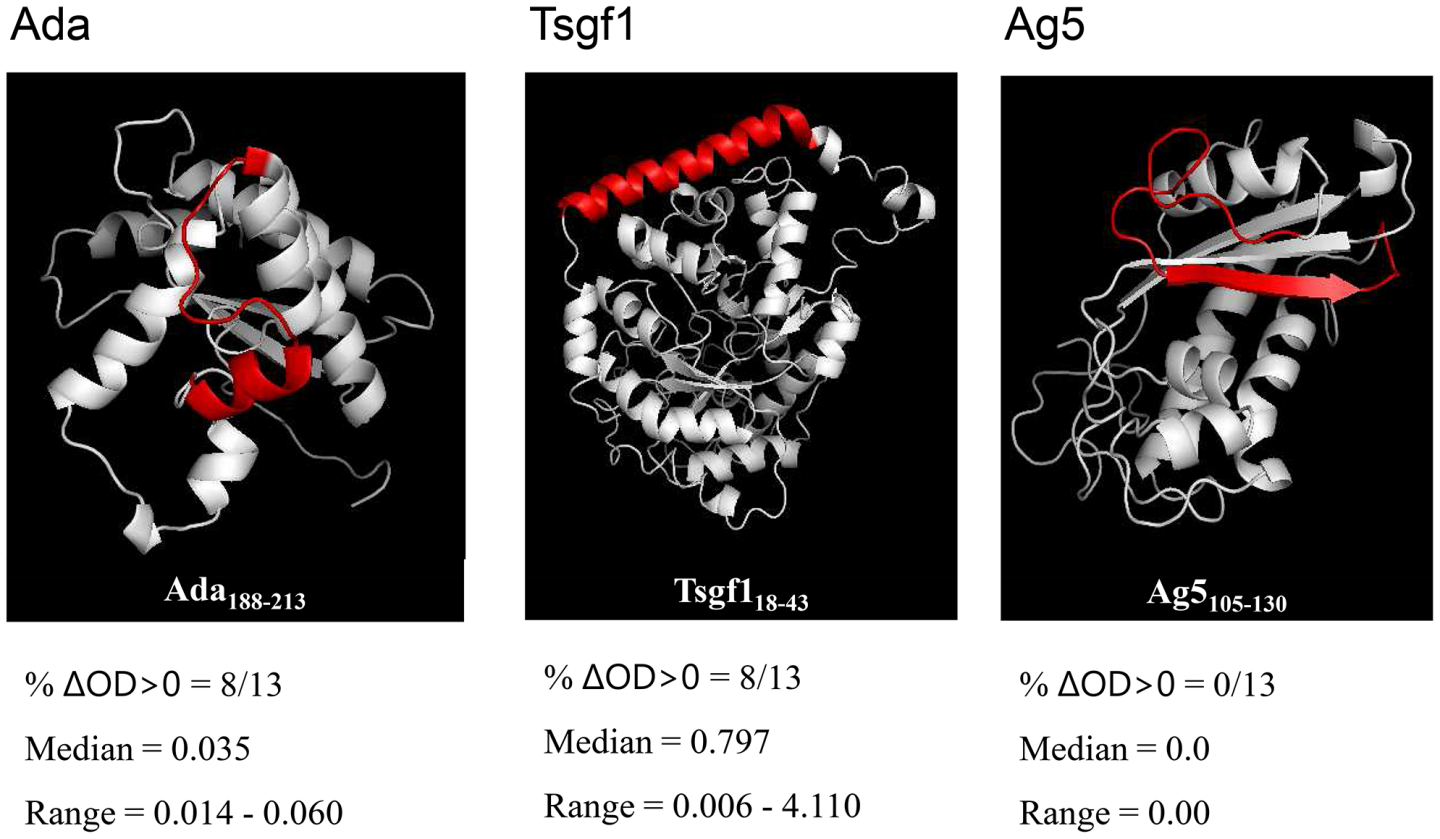
3D models of the Ada, Tsgf1, and Ag5 *Glossina* salivary proteins. 3D structure models were realized with Phyre and verified with molProbity server. The position of each selected candidate peptide is highlighted in red. Each peptide was evaluated in an ELISA indirect immunoassay against 13 plasma samples previously shown to react strongly with *Glossina palpalis gambiensis* whole saliva extracts [23]. Results are expressed in terms of number of plasma samples with ΔDO>0 and median and range of positive values for each synthetic peptide.

In a previous immune-proteomic approach combining 2-D electrophoresis and mass spectrometry, we were able to identify three proteins (Ada, Ag5 and Tsgf1), from *G. palpalis gambiensis* saliva extracts, that reacted specifically with IgG from individuals exposed to tsetse bites [23]. After the first screening step, where the sequence of each protein was submitted to four different epitope prediction algorithms, four to seven candidate epitopes (5 to 6 aa) were identified by at least three of the prediction algorithms for each of the study protein (Table 1). In a second step, candidate eptitope sequences (the 5–6 aa sequence added of 10 aa on each side) were submitted to Blast analysis with Genbank CDS non redundant databases. For each candidate sequence we calculated the difference between the Blast E-values for *G. morsitans morsitans* and the closest match in order to assess the specificity of candidate peptides to the *Glossina* genus (Table 1). For each protein, the peptide displaying the greatest difference were: Ada_188–213_ (*G. morsitans morsitans*, e = 3.10^−16^; *Drosophila melanogaster*, e = 5.6); Tsgf1_18–43_ (*G. morsitans morsitans*, e = 4.10^−15^; *Marinobacter algicola*, e = 1.3) and Ag5_105–130_ (*G. morsitans morsitans*, e = 2.10^−16^; *Vibrio mimicus*, e = 7.8). No relevant similarity was found with known human pathogens. After checking for peptide accessibility at the surface of the protein on 3-D models (Figure 1), Ada_188–213_, Tsgf1_18–43_ and Ag5_105–130_ were synthesized and tested on human plasma samples.

**Table 1:** Predicted antigenic peptides within the *Glossina morsitans morsitans* Ada, Tsgf1 and Ag5 salivary proteins.

Consensus peptides	E-value *G.m. morsitans*	Best match specie (E-value)
**ADA**		
**Seq1_71–96_**:	2.10^−16^	*Burkholderia pseudomallei* (0,96)
HLSTGGEVQL**NSKEIE**VDRIFMKYKI		
**Seq2_110–135_**	6.10^−17^	*Drosophila mojavensis* (5.10^−13^)
AAGMHFFKAK**PLIERS**KVFRFLQQMP		
**Seq3_168–193_**	6.10^−17^	*Leptospirillum ferrodiazotrophum* (1,7)
DQRGINILTF**RENPER**HKCTTQYVAV		
**Seq4_174–199_**	4.10^−17^	*Drosophila mojavensis* (0,0088)
ILTFRENPER**HKCTTQ**YVAVNEERQK		
**Seq5_188–213_**	**3.10^−16^**	***Drosophila melanogaster*** ** (5,6)**
TQYVAVNEER**QKSRSQ**ADYNRSFENL		
**Seq6_292–317_**	1.10^−15^	*Drosophila pseudoobscura pseudoobscura* (4,2)
DVLTVILEVV**GSFRSQ**YPDFLGVKLI		
**Seq7_315–340_**	1.10^−16^	*Arthrospira platensis str. Paraca* (2,3)
KLIYAINRRL**ETNEVR**NRVEILKKFQ		
**TSGF1**		
**Seq1_1–20_**	1.10–10	*Methylobacter tundripaludum* (0,17)
EVNKA**YQDER**NKILQEEEKL		
**Seq2_18–43_**	**4.10–15**	***Marinobacter algicola*** ** (1,3)**
EKLRLGYDVI**LQGDEE**KADQIFLRLK		
**Seq3_130–156_**	3.10–16	*Paramecium tetraurelia* (0,4)
DGCKSESVQV**SKGEAK**EDWFNLYTPT		
**Seq4_207–232_**	3.10–16	*Candida tropicalis* (0,71)
EVRVKFSEPY**DDTSKK**YSLDDVVKEI		
**Seq5_436–461_**	9.10–15	*Lactobacillus delbrueckii subsp. Bulgaricus* (0,4)
VENTIKYAQL**NSQEKT**AETLLKAKWN		
**AG5**		
**Seq1_14–39_**	2.10^−14^	*Daphnia pulex* (3)
VACVSQNVFQSGCSSDAKMIDLKKYQ		
**Sep2_105–130_**	**2.10^−16^**	***Vibrio mimicus*** ** (7,8)**
NLAELGRSGA**GTPDY**GQLIQKAVDS		
**Seq3_203–226_**	2.10^−13^	*Drosophila melanogaster* (1,7)
KSCSASAQDC**KTGKN**SKYQNLCSA		
**Seq4_221–243_**	1.10^−14^	*Stomoxys calcitrans* (0,008)
QNLCSANEKY**EVNRW**FKDGVEYQ		

All peptides that were identified by at least three out of four epitope prediction servers (NETSurfP, ABCpred, Bcepred and Proped-2) are listed for each protein. Within peptide sequences, amino acids forming the linear epitope are bolded. Each peptide was then blasted on all non-redundant Genbank CDS databases. Blast E-value for *G. morsitans morsitans* and the best match species are indicated. For each protein, the candidate peptide selected for biological validation is underlined.

### Peptide validation on human plasma samples

In order to assess antigenicity, each of the candidate peptide was evaluated in an ELISA immunoassay on a set of 13 plasma samples collected in tsetse-infested areas (Guinea and South West Burkina Faso) and displaying high IgG levels against *G. palpalis gambiensis* whole saliva extracts [23]. The highest IgG responses were observed for Tsgf1_18–43_ (8/13 samples with ΔDO>0; median of positive ΔDO = 0.79) whereas IgG responses appeared much weaker for Ada_188–213_ (8/13 samples with ΔDO>0; median of positive ΔDO = 0.03) or absent for Ag5_105–130_ (Figure 1). This last peptide was thus abandoned in further analysis.

**Figure 2:**
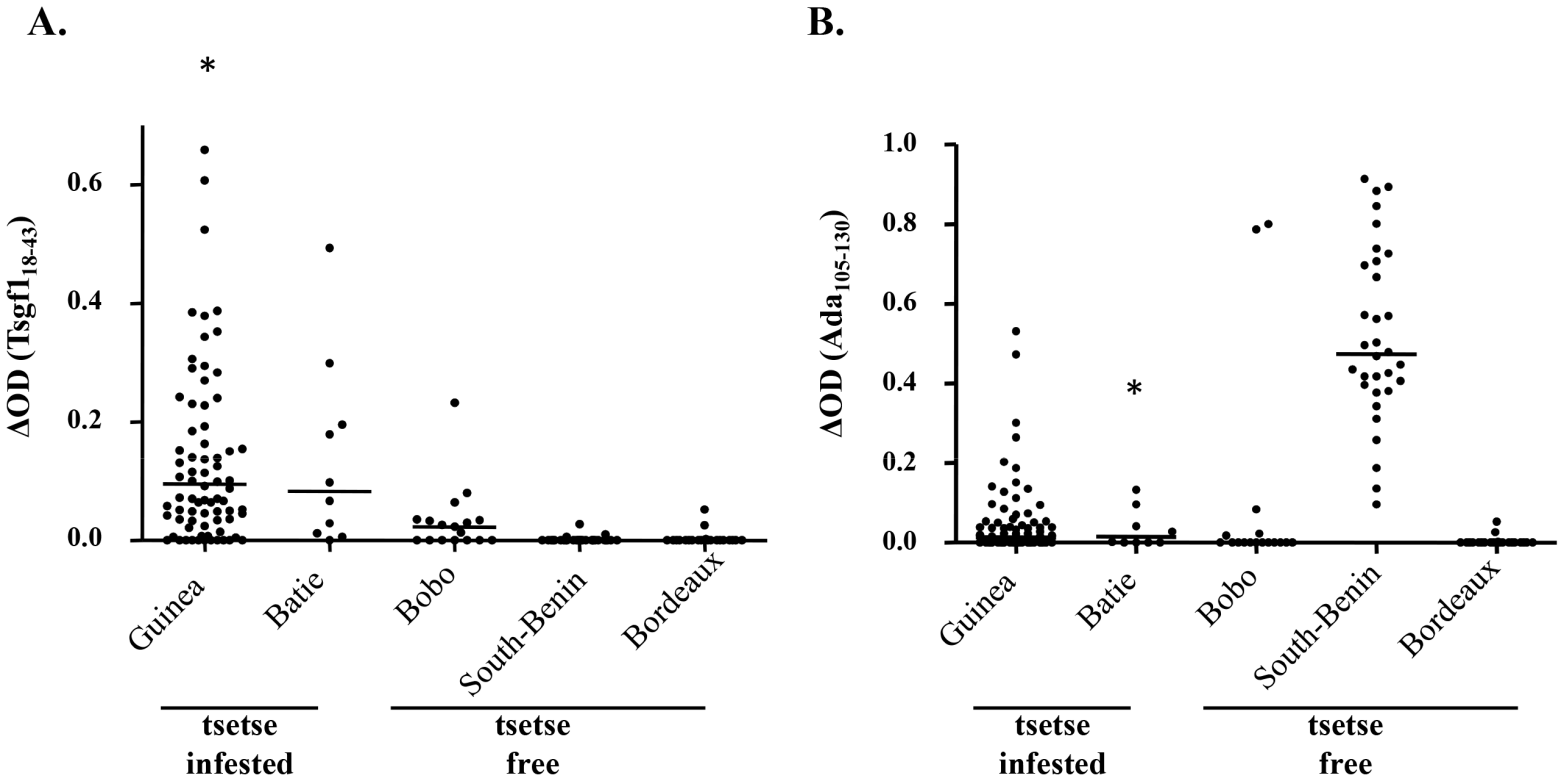
Tsgf1_18–43_ and Ada_188–213_ IgG responses in plasma samples from tsetse infested and tsetse free areas. IgG levels to Tsgf1_18–43_ (A) and Ada_188–213_ (B) were assessed by indirect ELISA in different study population from tsetse infested areas (Guinea; n = 80 and Batié; n = 10) and tsetse free areas (Bobo-Dioulasso; n = 17; South Benin; n = 31, and Bordeaux; n = 22). Asterisks “*” above the dot plots indicate that some values were higher than 0.65 for **A** (1.14, 1.21, 1.86, 3.10, 4.06 and 4.11) and higher than 1 for **B** (2.45). The horizontal bars in the graph represent the median of ΔDO values for each group.

The result of the ELISA immunoassay performed with Tsgf1_18–43_ and Ada_188–213_ on our sample of plasma collected from population exposed (Guinea, Batié) or not (Bobo-Dioulasso, South Benin and Bordeaux) to tsetse flies are shown in Figure 2. For Ada_188–213_ IgG responses were again low but were significantly higher in plasma from Guinea as compared to those from Bobo-Dioulasso and Bordeaux (p = 0.03). The same trend, although not significant, was observed for the sample from Batié (p = 0.15). Nevertheless strong immune responses to the Ada_188–213_ peptide were observed in almost all plasma samples from South Benin (the rural African control group) and two from Bobo-Dioulasso (the urban African control group) indicating potential cross-reactions for this candidate peptide. For Tsgf1_18–43_ a low level of IgG reactivity was observed in all tsetse-free areas (median ΔOD = 0.02 in Bobo-Dioulasso, and 0.00 in South Benin and Bordeaux). In contrast Tsgf1_18–43_ specific IgG levels were highly significantly increased in both tsetse-infested sites (Guinea, median ΔOD = 0.1, p<0.0001; Batie, median ΔOD = 0.08, p = 0.0001).

**Figure 3:**
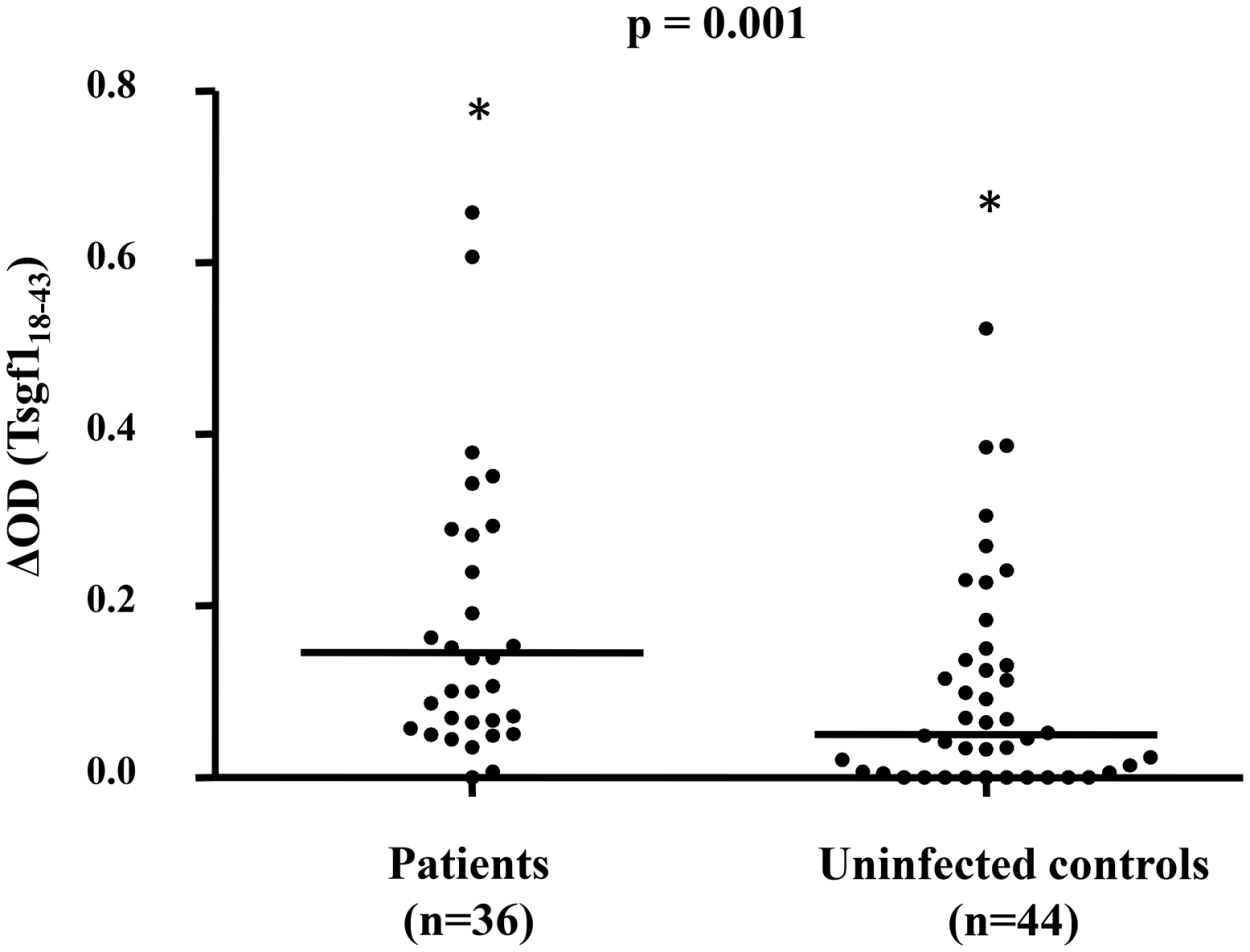
Anti-Tsgf1_18–43_ IgG levels in HAT patients and uninfected controls. This analysis only includes the plasma samples collected in the Guinean HAT foci. Thirty six samples were from parasitologically confirmed HAT patients and 44 were from uninfected controls from the same villages. The Mann Whitney test was used to compare Tsgf1_18–43_ specific IgG levels between the two groups. The horizontal bars in the graph represent the median of ΔDO values for each group. Asterisks “*” above the dot plots indicate that some values were higher than 0.8 in the patient group (4.11, 3.10, 1.85, 1.21, 1.14) and control group (4.06). The p-value of Mann Whitney U nonparametric test for the comparison between the two groups is given above the plot.

Finally Tsgf1_18–43_ was evaluated as a marker/indicator of the HAT risk in the Guinean disease foci (Figure 3) by comparing Tsgf1_18–43_ specific antibodies levels between HAT patients (n = 36) and *T. b. gambiense* uninfected controls (n = 44). HAT patients displayed significantly higher anti-Tsgf1_18–43_ specific antibody responses as compared to uninfected individuals (median ΔDO = 0.15 and 0.05 respectively, p = 0.001).

## Discussion

In a previous work, we identified three *G. palpalis gambiensis* salivary antigens (Ada, Ag5 and Tsgf1) that were specifically recognized by IgG antibodies from individuals exposed to tsetse flies in West Africa [23]. Here we report on the performance, as marker of exposure, of three synthetic peptides that were designed from these protein sequences by a bioinformatics approach and then tested on plasma from individuals naturally exposed or not to tsetse flies. We show that one of them (Tsgf1_18–43_) is a promising candidate to assess tsetse human contact in HAT foci.

Out of the three candidate peptides selected by the *in silico* approach, Ag5_105–130_ did not induce any response when tested with plasma samples that were shown to strongly react with *G. palpalis gambiensis* whole salivary antigens, and two appeared to be antigenic although responses observed with Ada_188–213_ were much weaker as compared to Tsgf1_18–43_. This was somehow an expected result, as the predictive value of the epitope prediction algorithms that were used in this study are known to be in the range of only 50 to 65% [42], [43]. Furthermore, for bioinformatics analyses we had to rely on protein sequences from *G. morsitans morsitans* as no *G. palpalis gambiensis* sequences are yet available. The low or absent antigenicity observed for Ada and Ag5 respectively may thus therefore result from sequence differences between *G. morsitans morsitans* and *G. palpalis gambiensis* to which our study population are exposed. Nevertheless, as the use of *G. morsitans morsitans* sequences may be regarded as a limitation to our study design, an advantage is also that the potential candidate peptide identified by our approach may be used not only to monitor *G. palpalis gambiensis* exposure in West Africa, but also exposure to *G. morsitans morsitans* and possibly other tsetse species in East and Central Africa.

In the next step of the analysis, we evaluated the specificity of the Tsgf1_18–43_ and Ada_188–213_ candidate peptides to detect exposure to tsetse flies by comparing specific IgG responses in plasma samples collected from a variety of populations from tsetse infested areas and tsetse free areas. Unexpectedly, all plasma samples from South Benin and 2 samples from Bobo-Dioulasso reacted strongly in the Ada_188–213_ ELISA immunoassay, evidencing that antibodies unrelated to tsetse exposure have the potential to induce cross reactions with this synthetic peptide. This is in contrast with the results of our Blast analysis that did not evidence any close matches to this peptide sequence. It is however clear that all sources of human antigens have not yet been sequenced and are thus not present in the data bases that were used for our “*in silico*” approach. Nonetheless, Tsgf1_18–43_ appeared to be a good candidate to assess human exposure to tsetse flies. Anti-Tsgf1_18–43_ IgG responses were low in all our control groups (from rural, urban Africa and France) and were highly and significantly elevated in plasma samples collected from our two populations from tsetse-infested areas (Guinea and SW Burkina Faso). Noteworthy an intermediate response was observed in one of the study control from Bobo-Dioulasso suggesting that anti-Tsgf1_18–43_ antibodies may not be entirely specific of tsetse exposure; we can however also not completely rule out that this subject was truly exposed to tsetse bites in the near neighborhood of the city. These results are similar to those previously reported when using *G. palpalis gambiensis* whole saliva extracts [23]. Importantly however, a higher proportion of intermediate responses was observed in individuals from tsetse-free areas when whole saliva extracts were used, thus suggesting a better specificity of Tsgf1_18–43_ to assess human exposure to tsetse flies. Furthermore, whereas no association had been observed between specific IgG levels directed against whole saliva extracts and the risk of HAT [23], anti-Tsgf1_18–43_ antibodies were more elevated in HAT patients as compared to uninfected individuals in the Guinean HAT foci. Here a possible explanation to this result could be again that examining plasma reactivity to a single epitope provides a better specificity to assess tsetse exposure as compared to whole saliva extracts. The saliva of blood sucking arthropod is composed of a complex mixture of proteins, some of which are common and conserved across species [50]. Use of whole salivary antigens may thus lead to potential spurious reactions impairing immunoassays. All together, these results suggest that anti-Tsgf1_18–_43 IgG antibodies could serve both as a direct biomarker of exposure to tsetse bites in West Africa and also as a marker to assess the *T. b. gambiense* infection risk in endemic areas. We were not able in the framework of this study to assess the dynamics of apparition and disappearance of *Glossina* saliva specific antibodies and the relation between IgG responses and the individual tsetse exposure levels. To this end, anti-Tsgf1_18–43_ IgG antibodies will be evaluated in sentinel villages from the Boffa focus in Guinea, before and after tsetse control intervention have been taken [7]. Noteworthy, we were able to show, that in cattle experimentally bitten by tsetse flies, *Glossina* saliva specific antibodies returned to pre-exposure levels within only few weeks after the stop of exposure (Somda et al., personnal communication).

Defining immunoassays that enable to overpass issues associated with the use of whole saliva extracts has been a major challenge to develop specific and sensitive tools to detect/measure host exposure to blood sucking arthropods. Resort to recombinant proteins has proved to be an efficient way to develop standardized immunoassays to a variety of disease vectors such as ticks, sanflies, triatomines or to Afrotropical malaria vectors [27], [28], [30], [31], [51]. Nevertheless, the production and the storage/shipment of recombinant proteins are often problematic and require good facilities which limit their use in many contexts such as in developing countries. On the contrary synthetic peptides are easy to produce and can be stored lyophilized. In this paper we have used an “*in silico*” approach to select candidate peptides within the sequence of our candidate proteins as experimental methods used for characterizing epitopes are time consuming and demand large resources. A similar approach has previously been successfully applied to design the gSG6-P1 peptide [33], [34], [52] from the gSG6 protein, a small anopheline-specific salivary protein [31]. In our study, out of only three synthetic peptides selected by bioinformatics tools, one was a good candidate characterized by a good antigenicity and specificity confirming further the validity of this approach. For logistic reasons we were only able to test three synthetic peptides, but other peptides identified within the Ada, Tsgf1 and Ag5 sequences (Table 1), with similar Blast estimated specificity to the *Glossina* genus, would now be interesting to test. Other salivary proteins could also be included as, although cross-reactive antibody may exist against the whole protein [23], some epitopes could be specific of the *Glossina* genus. Indeed whereas, as stated above, Tsgf1_18–43_ appears as a good candidate to assess exposure to tsetse flies providing a good specificity of the immunoassay, relying on only one epitope may impair sensitivity to detect anti-tsetse saliva antibodies as this specific epitope may not be recognized by all individuals exposed to tsetse bites. Indeed, in a recent study in which sera of mice experimentally bitten by *Phlebotomus papatasi* were tested against four different salivary recombinant proteins, it was shown that whereas each mice serum reacted with at least one of the recombinant protein, none of the recombinant proteins were recognized by all sera [53]. In a similar way it was shown that a combination of two *Lutzomia longipalpis* recombinant salivary antigens performed better than each of the individual ones to predict human anti-salivary gland sonicate positivity [28].

In this paper, we have shown that the analysis of plasma reactivity to the Tsgf1_18–43_ synthetic peptide provides an easy and cheap way to monitor human exposure to tsetse flies in West Africa and in contrast to whole saliva extracts to assess HAT risk in endemic populations. Combining such a serological test with the mass screening Card Agglutination Test for Trypanosomiasis [54], performed on thousands of individuals during medical surveys, could provide National Control Programs with important indicators to quickly map human exposure levels in order to better target vector control efforts and to monitor the efficiency of vector control campaigns to lower human tsetse contacts. Finally, we have confirmed that *in silico* approaches represent interesting and useful tools to design synthetic peptide to assess human exposure to arthropod bites. Inclusion of other *Glossina* salivary antigens and biological validation of other predicted antigenic peptides identified in the framework of this study should thus enable to improve further the sensitivity of anti-*Glossina* saliva specific IgG detection by allowing the design of peptide cocktail based immunoassay.
